# Esophageal Squamous Papilloma: An Unusual Cause of Dysphagia

**DOI:** 10.7759/cureus.109191

**Published:** 2026-05-19

**Authors:** Sneha Adidam, Temitayo Gboluaje, Nakul Ganju, Naga Sai Shravan Turaga, Srikanth Adidam Venkata, Farshad Aduli

**Affiliations:** 1 Gastroenterology, Howard University Hospital, Washington, D.C., USA; 2 Gastroenterology and Hepatology, First State Gastroenterology, Dover, USA; 3 Internal Medicine, Howard University Hospital, Washington, D.C., USA; 4 Cardiology, AdventHealth Florida Hospital, Altamonte Springs, USA; 5 Neurology, Downstate Neurology at One Brooklyn Health, New York, USA

**Keywords:** cold biopsy forceps, dysphagia, esophageal polyp, esophageal squamous papilloma, esophagogastroduodenoscopy, helicobacter pylori

## Abstract

Esophageal squamous papilloma (ESP) is a rare benign epithelial tumor of the esophagus with an endoscopic prevalence of 0.01% to 0.43%. It is most commonly discovered incidentally during upper endoscopy in asymptomatic patients. Symptomatic presentation, particularly dysphagia, is uncommon and infrequently reported in the literature. We present the case of a 58-year-old female with a three-month history of progressive dysphagia to both solids and liquids, along with intermittent epigastric pain. Esophagogastroduodenoscopy (EGD) revealed a solitary 3 mm sessile polyp in the middle third of the esophagus, which was successfully resected using cold biopsy forceps. Histopathology confirmed ESP, demonstrating papillary proliferation of non-dysplastic stratified squamous epithelium with fibrovascular cores, variable parakeratosis, spongiosis, and mild basal cell hyperplasia. Following resection, the patient experienced complete resolution of her dysphagia. Concurrently, gastric biopsies revealed active *Helicobacter pylori *(*H. pylori*) gastritis, for which standard triple eradication therapy was initiated. This case highlights ESP as a rare but clinically important etiology in the workup of unexplained dysphagia, demonstrates the curative potential of endoscopic resection even for small lesions, and reinforces the need for awareness of this entity among clinicians evaluating upper gastrointestinal symptoms.

## Introduction

Esophageal squamous papilloma (ESP) is an uncommon benign neoplasm of the esophageal mucosa, with a reported endoscopic prevalence ranging from 0.01% to 0.43% [1]. Its pathogenesis remains incompletely understood, though several contributing factors have been proposed, including human papillomavirus (HPV) infection, chronic mucosal irritation secondary to gastroesophageal reflux disease (GERD), and mechanical trauma from devices such as esophageal metal stents [2]. Histologically, ESP is characterized by papillary fronds of non-dysplastic squamous epithelium overlying fibrovascular cores of the lamina propria, often accompanied by parakeratosis and basal cell hyperplasia [3]. The overwhelming majority of ESPs are asymptomatic and identified incidentally on upper endoscopy performed for unrelated indications - lesions are typically small, often measuring less than 5 mm, and not ordinarily expected to produce obstructive symptoms. When symptoms are present, dysphagia and epigastric discomfort are among the most commonly reported; however, symptomatic ESP remains a rarely documented clinical scenario, particularly when attributable to a diminutive, solitary lesion of this size. The possibility of malignant transformation, while uncommon, has been reported, lending clinical significance to accurate diagnosis and appropriate endoscopic management [4,5].

Herein, we present a case of a 58-year-old female who presented with a three-month history of progressive dysphagia and was found to have a solitary ESP in the middle esophagus on esophagogastroduodenoscopy (EGD). Complete endoscopic resection resulted in full symptomatic resolution. This case underscores the importance of including ESP in the differential diagnosis of dysphagia and highlights the diagnostic and therapeutic role of upper endoscopy.

## Case presentation

A 58-year-old female was referred to the gastroenterology clinic for evaluation of progressive dysphagia. She described a three-month history of intermittent difficulty swallowing both solids and liquids, associated with intermittent epigastric pain. She denied odynophagia, hematemesis, melena, unintentional weight loss, and symptoms of heartburn or acid regurgitation. Her past medical history was significant for a personal and family history of peptic ulcer disease. Social history was notable for active tobacco use (two packs per day) and occasional marijuana use (approximately twice per month). She denied alcohol use.

On physical examination, vital signs were within normal limits. Body mass index (BMI) was 32 kg/m². Abdominal examination revealed mild diffuse tenderness on deep palpation without guarding, rigidity, or rebound tenderness. The remainder of the examination was unremarkable. Laboratory investigations, including a complete blood count and comprehensive metabolic panel, were within normal limits. No imaging was obtained prior to endoscopy.

**Figure 1:**
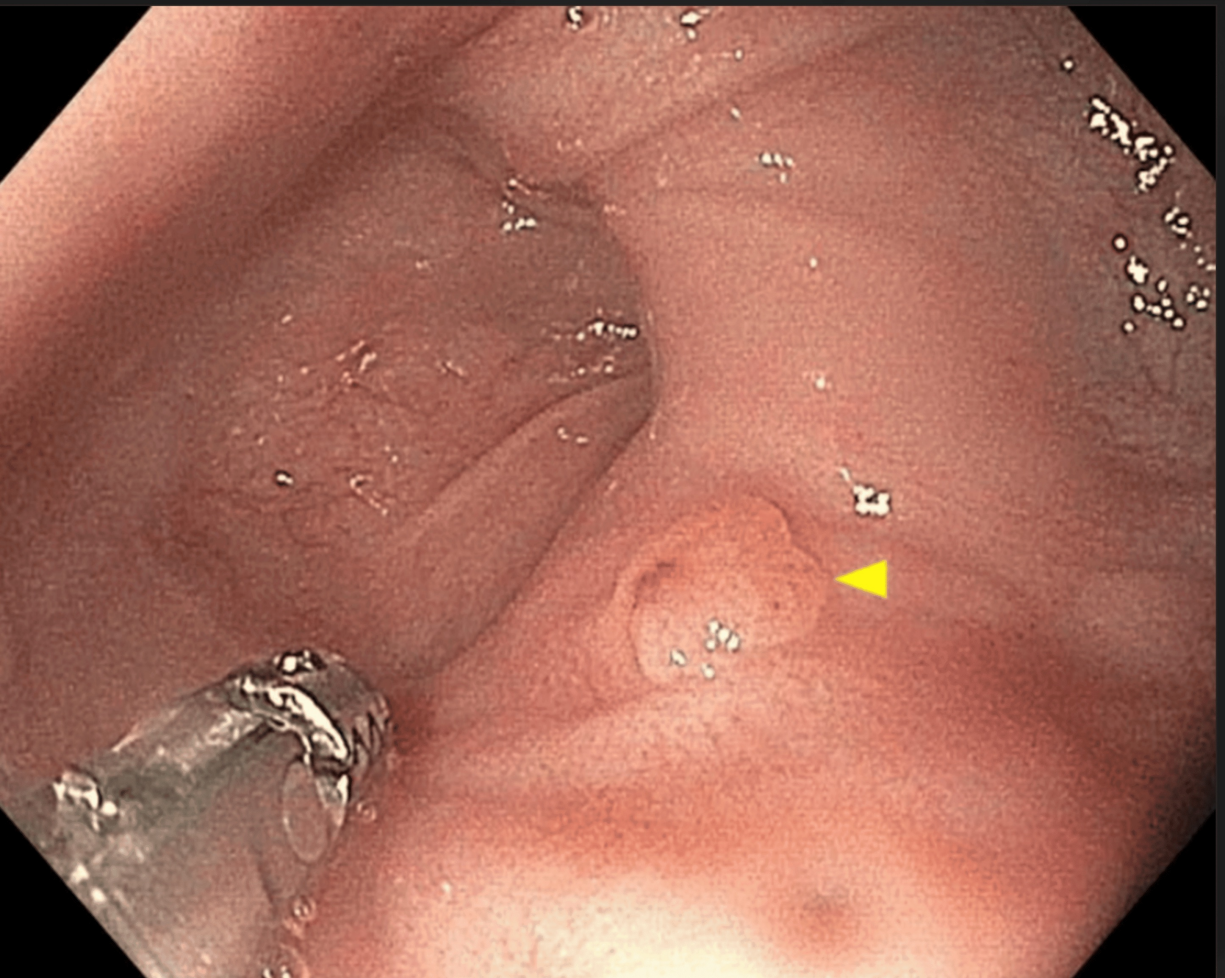
Esophagogastroduodenoscopy demonstrating a single 3 mm sessile polyp in the middle third of the esophagus (yellow arrowhead).

**Figure 2:**
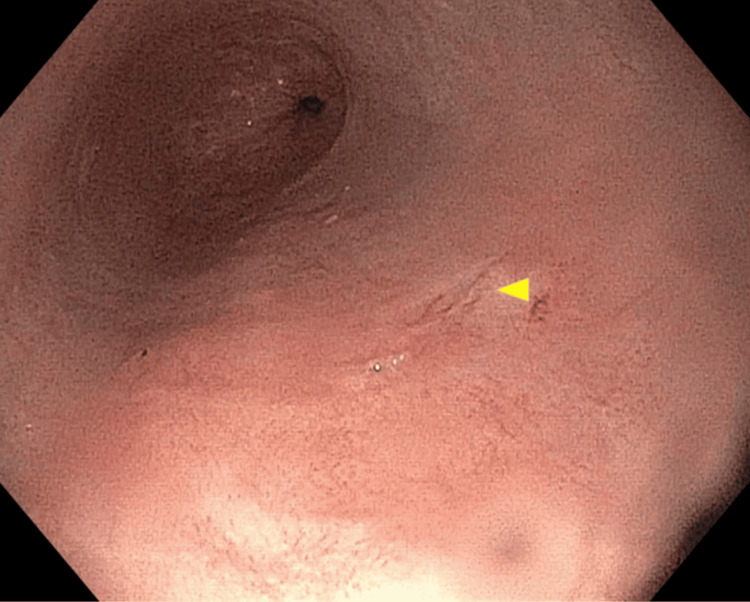
Post-polypectomy site following cold biopsy forceps resection (yellow arrowhead).

The patient underwent esophagogastroduodenoscopy (EGD), which revealed a single 3 mm sessile polyp in the middle third of the esophagus (Figure 1). The remainder of the esophageal mucosa appeared grossly normal. The lesion was resected in its entirety using cold biopsy forceps under direct endoscopic visualization, with a satisfactory post-polypectomy site confirmed (Figure 2, arrowhead). Simultaneous random biopsies were obtained from the gastric antrum and body.

**Figure 3:**
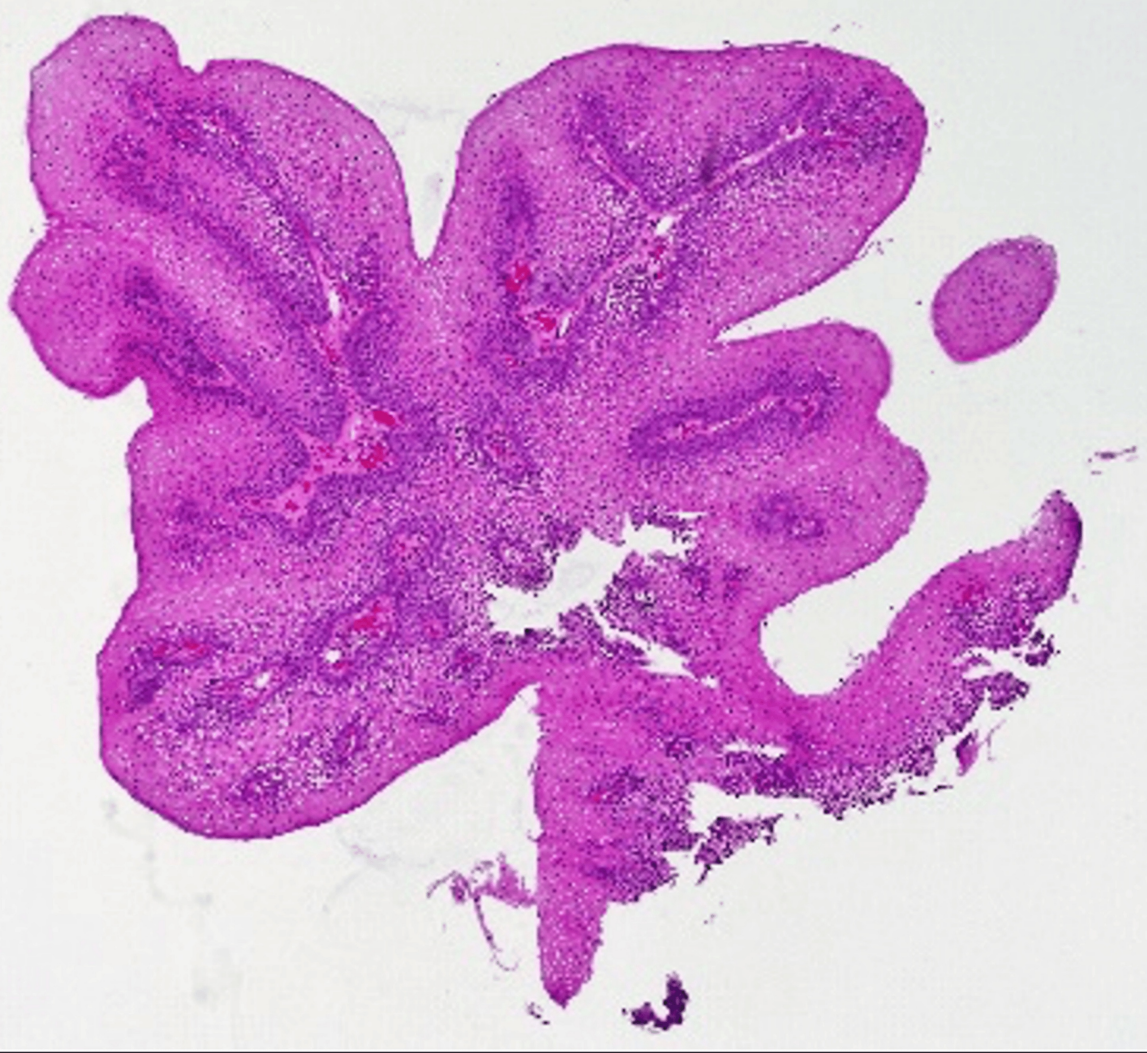
Histopathology demonstrating papillary proliferation of stratified squamous epithelium with variable parakeratosis (hematoxylin and eosin stain, x20).

**Figure 4:**
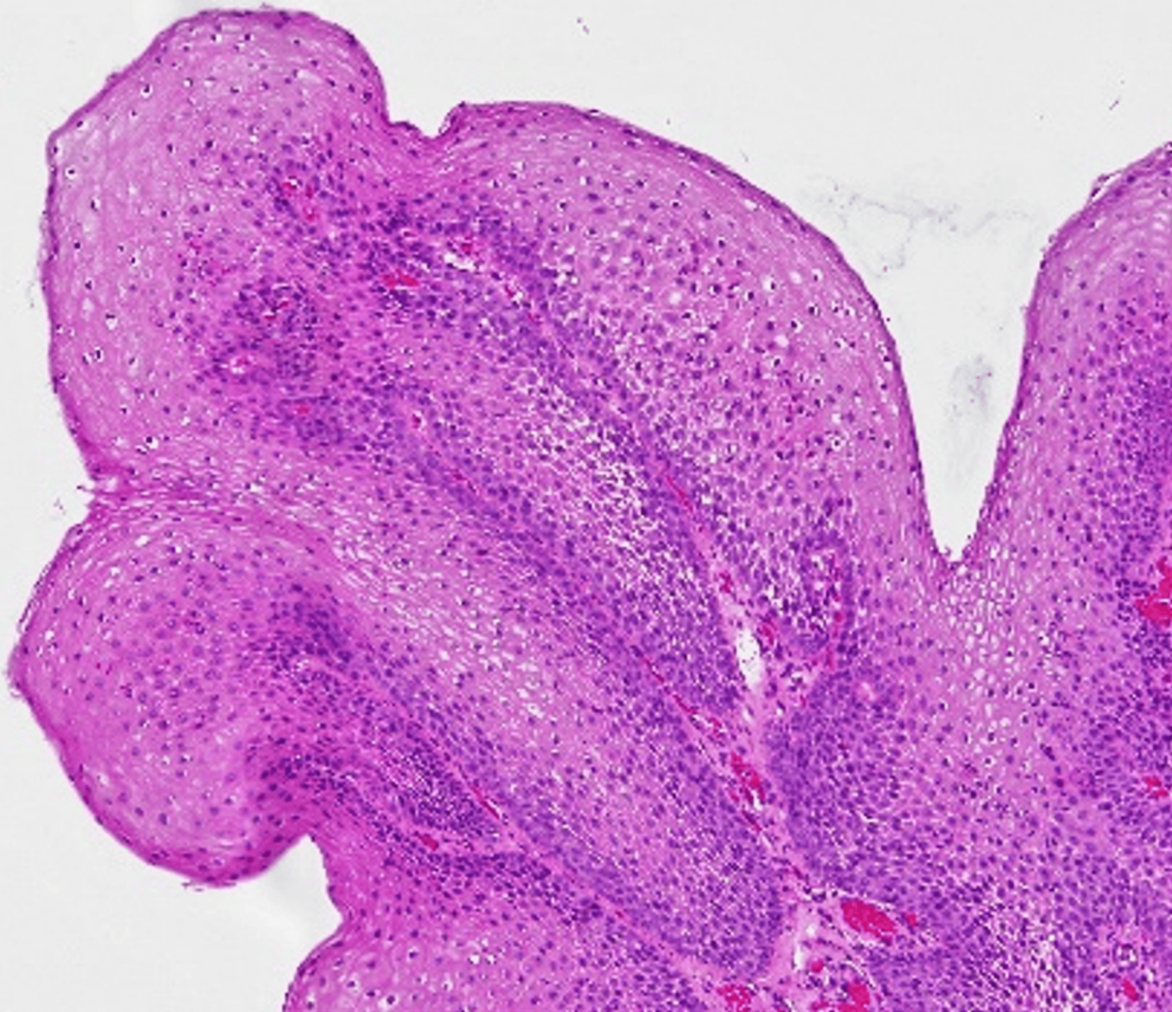
High-power histopathology showing finger-like projections with prominent fibrovascular cores, spongiosis, and mild basal cell hyperplasia (hematoxylin and eosin stain, ×40)

Histopathological examination of the esophageal specimen demonstrated papillary proliferation of stratified squamous epithelium with variable parakeratosis (Figure 3) and finger-like projections with prominent fibrovascular cores, associated spongiosis, and mild basal cell hyperplasia (Figure 4). No dysplasia, atypia, or features of malignancy were identified. These findings were diagnostic of esophageal squamous papilloma. Gastric biopsies revealed active gastritis with confirmed *Helicobacter ​​​​​pylori *(*H. pylori*)* *organisms.

At the post-procedure follow-up visit, the patient reported complete and sustained resolution of her dysphagia. Persistent epigastric pain was attributed to *H. pylori*-associated gastritis, and she was initiated on standard triple therapy consisting of a proton pump inhibitor, clarithromycin, and amoxicillin for 14 days. The patient was subsequently lost to follow-up, and test-of-cure could not be confirmed.

## Discussion

Esophagogastroduodenoscopy is a rare benign epithelial tumor most commonly identified incidentally on upper endoscopy. It predominantly affects middle-aged individuals and has a slight male predominance in most reported series [6]. Lesions are typically solitary, small (less than 5 mm), and sessile with a warty or verrucous surface morphology. The majority arise in the distal third of the esophagus, a distribution that supports the hypothesis of chronic acid reflux and mucosal irritation as contributing pathogenic mechanisms [6]. Our patient's lesion was located in the middle third of the esophagus - a less common location that has been more frequently associated with HPV-related ESPs in prior studies, as HPV-positive lesions tend to favor proximal and middle esophageal locations compared to their HPV-negative counterparts [7]. HPV testing was not performed in this case, representing a limitation; its inclusion in future cases may further elucidate the relationship between viral etiology and anatomic distribution.

Clinically, the presentation of ESP is highly variable. The majority of patients are asymptomatic; Quitadamo and Benson reported that more than 15% of patients with ESP had no gastrointestinal complaints at the time of diagnosis [8]. When symptoms do occur, dysphagia and epigastric discomfort are most commonly described. What makes this case particularly instructive is the direct causal relationship demonstrated between a small (3 mm), solitary ESP and clinically significant dysphagia to both solids and liquids - a symptom burden that completely resolved following endoscopic resection. This outcome illustrates that even diminutive lesions in critical anatomic locations can produce meaningful obstructive symptoms, and that symptom severity alone should not preclude endoscopic evaluation for this entity.

Histopathologically, our case exhibited the classic features of ESP: papillary proliferation of non-dysplastic stratified squamous epithelium, prominent fibrovascular cores within finger-like projections, variable parakeratosis, spongiosis, and mild basal cell hyperplasia, all without evidence of dysplasia or malignant transformation [6]. These findings are consistent with previously published histologic descriptions and support the benign nature of this lesion in its solitary form.

Regarding management, multiple endoscopic modalities have been described for ESP resection, including cold biopsy forceps, snare polypectomy, endoscopic mucosal resection (EMR), photodynamic therapy, and radiofrequency ablation [8,9]. No evidence-based guidelines currently establish the superiority of one technique over another, and selection is guided by lesion size, morphology, and endoscopist preference. In this case, cold biopsy forceps resection was appropriate, given the small size and solitary nature of the lesion, and yielded both complete histologic diagnosis and durable symptom resolution.

The risk of malignant transformation in ESP, while low, is not negligible. Progression to esophageal squamous cell carcinoma has been documented in the setting of larger lesions, multiple papillomas, and papillomatosis [5,10]. Given this potential, post-resection endoscopic surveillance is advisable, though evidence-based intervals have not been formally established. In our case, the patient was lost to follow-up, highlighting a practical challenge in ensuring appropriate long-term monitoring in this population.

Finally, the concurrent finding of *H. pylori*-positive active gastritis in this patient is a noteworthy incidental discovery. While *H. pylori *is not implicated in the pathogenesis of ESP, its detection and treatment carry independent clinical importance given the patient's personal and family history of peptic ulcer disease. This case demonstrates the value of comprehensive endoscopic evaluation - including systematic gastric biopsies - in patients presenting with upper gastrointestinal symptoms, even when a primary esophageal etiology is identified.

## Conclusions

This case demonstrates that ESP, though typically asymptomatic, can present as the primary etiology of clinically significant dysphagia, even when the lesion is small and solitary. Endoscopic resection is both diagnostic and curative. Clinicians should include ESP in the differential diagnosis of unexplained dysphagia to avoid delay in diagnosis and treatment. Given the reported potential for malignant transformation with larger or multiple lesions, post-resection surveillance is recommended. Comprehensive endoscopic evaluation, including gastric biopsies, should be performed in all patients with upper gastrointestinal symptoms to identify concurrent pathology.
